# Portuguese students' knowledge of antibiotics: a cross-sectional study of secondary school and university students in Braga

**DOI:** 10.1186/1471-2458-9-359

**Published:** 2009-09-23

**Authors:** Maria Manuel Azevedo, Céline Pinheiro, John Yaphe, Fátima Baltazar

**Affiliations:** 1 School E.B.2, 3 D. Maria II, Vila Nova de Famalicão Portugal, Portugal; 2 Life and Health Sciences Research Institute (ICVS), School of Health Sciences, University of Minho, Campus de Gualtar, 4710-057 Braga, Portugal; 3 Community Health, School of Health Sciences, University of Minho, Campus de Gualtar, 4710-057 Braga, Portugal

## Abstract

**Background:**

Recent surveys show that the knowledge of the general public about the correct use of antibiotics is limited. This contributes to the problem of inappropriate antibiotic use, leading to a progressive loss of bacterial sensitivity to these drugs and the spreading of resistant strains of bacteria.

**Methods:**

In this cross-sectional study, a questionnaire about antibiotic use was given to a sample of students in the 9^th ^and 12^th ^grades of secondary school and in the first year of university in the north of Portugal.

**Results:**

349 students returned completed questionnaires. Deficits were found in the students' knowledge of antibiotics and their correct use. Only 4% of 9^th ^grade students were aware that antibiotics are used to treat bacteria only, while 14% of 12^th ^grade students and 29% of first-year university students were aware of this. Fewer students were aware that antibiotics are used to treat tuberculosis. There were deficiencies in the knowledge of timing and duration of therapy. However close to 70% of these students are aware that inappropriate use of antibiotics can contribute to resistance to these drugs.

**Conclusion:**

This study has observed a lack of general knowledge on correct antibiotic use in Portugal, as has been found in other countries. Since this may be due to a lack of formal education on this subject, we believe that a teaching unit on infectious diseases should be included in the 9^th ^and 12^th ^grades, in all curricular areas, with emphasis on bacterial and viral pathogens and correct antibiotic use. In addition, education on the correct use of medications may need to begin at much earlier ages.

## Background

Antibiotics are among the most commonly prescribed medications, however they are very often misused [1,2]. Among other factors, the indiscriminate use of antibiotics has contributed to the progressive loss of bacterial sensitivity to antibiotics and spreading of resistant strains of bacteria [3], with substantial clinical and economic impact [4]. The clinical effectiveness of antibiotics depends partially on their correct use, depending on patients, physicians and retailers [5]. Physicians' decisions may be influenced by several factors such as the fear of losing a patient's trust, the lack of correct information on indications for antibiotic use and pressure from patients and families [2]. Patient factors relating to incorrect antibiotic use include self-medication, sharing medication with other people, not taking a full course of treatment and keeping part of the course for another occasion [2,6].

A study performed in 2001 by Eurobarometer revealed that 60% of Europeans do not know that antibiotics are ineffective against viruses [7]. This lack of accurate information may result in high rates of inappropriate consumption. In 2002, Davey and collaborators [8] reviewed several examples of misperceptions regarding antibiotic use in respiratory tract infections. A study by Pechere in 2001 [9], carried out in different countries with more than 5,000 individuals, reported that more than 60% of those studied believed that antibiotics should be prescribed for viral illnesses. Other surveys showed that most of the patients with acute respiratory symptoms expect to receive antibiotics [10-12]. Another study showed that, in Moldova, many adults are unaware that antibiotics do not cure viral infections and the authors believe that many physicians and pharmacists dispense and prescribe antibiotics without regard to the cause of infection [13].

In Portugal, as most countries, antibiotics are dispensed only by prescription however, studies show that people can still obtain antibiotics without prescriptions [9]. McKee found that 26% of patients studied in his sample of American patients had obtained antibiotics without a physician's prescription and this has been observed in European countries as well [14,15].

Even when antibiotics are available only by prescription, the education of the public on correct use of antibiotics is necessary for the success of the treatment and prevention of the spread of bacterial resistance [16]. Information campaigns on antibiotic resistance carried out in Belgium, led to important reduction in antibiotic consumption [17]. Despite the importance of public information campaigns, the effect of an individual campaign is transient, thus these interventions must be sustained.

Following analysis of the Portuguese curriculum in Natural sciences and Biology, it was noted that very little teaching is done in the field of Microbiology. In the 6^th ^grade, among 11 and 12 year-old students, there is a teaching unit on Microbiology. However, the program is limited to microbial classification, characteristics of microorganisms, pathogenicity and control of microbial growth. In later years there are no other courses on Microbiology.

Given the lack of correct information on antibiotics and its association with antibiotic resistance, an evaluation was planned to test the knowledge of Portuguese students in the 9^th ^grade (14 to 15 years old), 12^th ^grade (16 to 17 years old), and first-year university students in different fields of study, regarding antibiotic spectra, indications and their correct use. Differences in the students' knowledge of this subject by grade level and the area of study were to be assessed.

## Methods

This survey was conducted between February and April 2007. A convenience sample was used comprising 349 students including 179 from the 9^th ^and 12^th ^grades and 170 first year university students from the district of Braga, the third largest city in Portugal. The sample of 9^th ^year students comprises 80% of the total number of 9^th ^grade students from D. Maria II School, V. N. de Famalicão. In the 12^th ^year, one class was selected at random from each study area from secondary schools in the same city. The sample of first year university students comprises all students from the selected courses taught by the University of Minho (Braga). Ethical approval for the study was obtained from the directors of the schools involved in the study. Participation in the study was voluntary and anonymous. Participants were informed of this and assured that no participant could be identified from pooled presentation of the results. There were no refusals to participate. The data were obtained through the administration of a seven-item questionnaire [see additional file 1], developed by the authors and pre-tested with a sample of 10 subjects for comprehension of the questions. Questionnaires were completed during regular classes with a time limit of 20 minutes for completion. The questionnaire was designed to assess the student's knowledge on the types of organisms sensitive to antibiotics, the types of infectious diseases treated with antibiotics and the correct use of antibiotics.

Data were analysed using the SPSS statistical software Version 14.0. (SPSS Inc. Chicago, IL, USA). Associations between variables were tested with Pearson's Chi-square (χ2) with significance set at the p < 0.05 level.

## Results

The response rate was 100% among the 349 students asked to participate. The characteristics of the study population are shown in Table 1.

**Table 1:** Characteristics of the study population (n = 349).

**Grade level**	**Age (years)**	**Number of students**
**9^th ^grade**	14-18	100

**12^th ^grade**		79
Science	16-17	39
Humanities	16-18	7
Informatics	16-19	19
Arts	16-20	14

**1^st ^year of university**		170
Nursing	18-20	65
Biology/Geology	18-21	14
Law	18-22	49
Engineering	18-22	42

Data presented in Table 2 show the knowledge of students on the sensitivity of organisms to antibiotics. There is an increase in the number of correct answers by grade level up to university, however with significant heterogeneity in answers among university study areas. The highest scores were obtained by students in the study areas with closer affinity to the topic assessed, such as sciences (12^th ^grade) and nursing (university).

**Table 2:** Percentage of positive answers to question on antibiotic use against bacteria and other organisms.

***Options***	**n**	Antibiotics are effective against bacteria only * (%)	**Antibiotics are effective against bacteria and other organisms (%)**	**Antibiotics are effective against other organisms (%)**	***P value***
**9^th ^grade**	100	4	65	31	

**12^th ^grade**					

*Sciences*	39	28	46	26	
*Humanities*	7	14	14	72	
*Informatics*	19	0	74	26	
*Arts*	14	14	65	21	
Mean ± SD		14 ± 11.4	49.8 ± 26.5	36.3 ± 24	0.066

**1^st ^year of university**					

*Biology/Geology*	14	29	57	14	
*Nursing*	65	60	34	6	
*Law*	49	16	57	27	
*Engineering*	42	14	62	24	
Mean ± SD		29.8 ± 21.2	52.5 ± 12.6	17.8 ± 9.6	<0.001

SD-Standard deviation, *- Correct answer

The students' knowledge of the value of antibiotics in the treatment of common diseases of different aetiologies is presented in Table 3. The number of correct answers increases with the grade level, with marked heterogeneity among study areas in 12^th ^grade. There are significant differences between the answers of students from different study areas (12^th ^grade and university). The highest scores were obtained by students in the sciences (12^th ^grade) and nursing (university). There are high percentages of incorrect answers among all grade levels evaluated.

**Table 3:** Percentage of positive answers to questions on antibiotic use against viral illness (influenza, hepatitis, AIDS) and tuberculosis.

***Options***	**n**	Antibiotics should be prescribed for tuberculosis * (%)	**Antibiotics should be prescribed for tuberculosis and viral illnesses (%)**	**Antibiotics should be prescribed for viral illnesses (%)**	***P value***
**9^th ^grade**	100	2	3	95	

**12^th ^grade**					

*Sciences*	39	18	8	74	
*Humanities*	7	0	14	86	
*Informatics*	19	0	38	62	
*Arts*	14	0	28	72	
Mean ± SD		4.5 ± 9.0	22 ± 13.6	73.5 ± 9.9	0.049

**1^st ^year of university**					

*Biology/Geology*	14	14	43	43	
*Nursing*	65	38	30	32	
*Law*	49	4	19	77	
*Engineering*	42	13	19	68	
Mean ± SD		17.3 ± 14.6	27.8 ± 11.4	55 ± 21.0	0.001

SD-Standard deviation, *- Correct answer

The responses to questions on correct antibiotic use are shown in Table 4. Significant differences were found in the proportion of correct responses among the students from the three grade levels assessed, with an increase in the number of correct answers by grade level. The highest scores were obtained by students in the study areas with closer affinity to the topic assessed.

**Table 4:** Percentage of correct answers to questions on antibiotic treatment for bacterial infections.

		**Grade 9 (%)**	**Grade 12** **(%)**	**1^st ^year university (%)**	
**n**	**Correct answer**	**100**	**79**	**170**	

**Study question**			**Mean ± SD ^a^**	**Mean ± SD^b^**	**Overall *p***

Antibiotics should be taken with milk	F	57	51.8 ± 13.5	59.2 ± 10.1	0.537
Antibiotics do not interact with alcohol	F	46	65.5 ± 6.6	75.0 ± 11.3	<0.001
Antibiotics can be taken at different times each day, if the daily doses are taken.	F	50	49.5 ± 21.9	76.0 ± 9.9	<0.001
Antibiotic treatment should be stopped as soon as the patient feels better	F	57	66.0 ± 20.4	77.7 ± 8.8	<0.001
Remaining antibiotic doses can be saved for use on other occasions	F	59	57.5 ± 11.1	79.2 ± 10.7	<0.001
The incorrect use of antibiotics can lead to development of resistant bacteria.	T	70	68.0 ± 17.8	77.2 ± 5.3	0.570

SD-Standard deviation; T = true; F = false.

^a^, mean score from 4 study areas: sciences, humanities, informatics and arts

^b^, mean score from 4 study areas: biology/geology, nursing, law and engineering

## Discussion

This study assessed the knowledge of antibiotics among Portuguese school students of 9^th ^and 12^th ^grade and first year of university. We believe this was the first study of its kind to be performed in Portugal.

A convenience sample of high-school and university students was used to allow for rapid collection of data in a short period of time with limited resources. Information about possible confounders, such as socio-economic status and intellectual level, was not collected. These factors may limit the generalizability of our findings. Repeating this study in other areas of the country with random sampling of students and attention to possible confounders will help answer this question.

Students' knowledge on antibiotic spectra and indications for use were limited at all three school levels evaluated, and misconceptions were prevalent among students, however with a lower error rate for nursing students. The differences observed in scores among 12^th ^grade science students, nursing students and the remaining students, could be explained by either acquisition of knowledge from different sources other than school, or the result of selection of students, given the higher marks required for admission to science higher degrees.

With respect to correct antibiotic use, our results showed limited knowledge among 9^th ^and 12^th ^grade students and nursing students obtained the best results among university students. This issue is of concern because many students complete their schooling after 9 years of compulsory education and may receive no further instruction on this topic. This may also influence subsequent generations. Recent studies showed that mothers often influence medical decisions on antibiotic prescription [13]. In this context, paediatricians are often pressured to prescribe antibiotics to children with viral infectious [18,19]. Cebotarenco [20] showed that in times of high incidence of viral infection, half of these infectious diseases are treated with antibiotics, self prescription. In addition, mothers may administer these drugs to children without medical advice [13]. This suggests the need for more widespread education on the proper use of antibiotics. An extensive school-based educational program in Moldova, which included peer-education sessions, parents' meetings, and distribution of educational newsletters, was successful in reducing antibiotic use for treatment of presumed viral respiratory illnesses [13]. Bush has suggested that these efforts begin as early as kindergarten based on observations of successful programs involving young children and their parents [21]. The United States Pharmacopeia Ad Hoc Advisory Committee on Children and Medicines has guidelines for preparation of educational materials about medication for children as young as three years old. As outlined in the USP Guiding Principles for teaching children and adolescents about medication, education needs to be tailored to their development, capabilities and experience [22].

In the present study, there was a trend towards an increase in the number of correct answers with the grade level. This is in accordance with the study of You and co-workers [23], who suggested that higher education is a positive predictor for adequate knowledge and appropriate attitudes to antibiotic use. Increasing prevalence of antibiotic resistance by bacteria, partly due to indiscriminate widespread use of antibiotics, is a threat to public health. Increasing public awareness of the problem and education of the general public and retailers on proper usage of antibiotics may help to slow this trend [24,25]. Patient demand for antimicrobials might be triggered by mass media, such as TV, internet, magazine or newspaper advertising, behaviours which also contribute to the development of resistance. In a study undertaken in Europe in 1997, physicians elected patients' pressure as the main reason for prescribing inadequate antibiotics [2]. These findings highlight the need to educate people about the appropriate use of antibiotics.

## Conclusion

There were marked deficiencies found in the knowledge of Portuguese students of antibiotics and their correct use. This may be due to a lack of formal education on the subject in schools. Education about the correct use of medication may need to begin at very early ages. We believe that a teaching unit on Microbiology should be included in 9^th ^grade curriculum, with emphasis on knowledge of antibiotic spectra/indications and correct antibiotic use. This unit should be reinforced in the 12^th ^grade in all curricular areas. We also believe that it is important to design teaching programs to be tested in schools to improve the knowledge of students on this subject. We suggest that this approach may modify behaviours with regard to antibiotic use with benefits for public health.

## Competing interests

The authors declare that they have no competing interests.

## Authors' contributions

MMA and FB designed the study and wrote the manuscript. CP performed data analysis and helped write the manuscript. JY helped to write and edit the manuscript. All authors have read and approved the final version of the manuscript.

## Pre-publication history

The pre-publication history for this paper can be accessed here:



## Supplementary Material

Additional file 1 **Questionnaire**. This file contains the questionnaire given to the students concerning antibiotic use. Click here for file
