# Supplementary material for: Portuguese students' knowledge of antibiotics: a cross-sectional study of secondary school and university students in Braga
Source: BMC Public Health. 2009 Sep 23;9:359. doi: 10.1186/1471-2458-9-359 (PMC2759931; doi:10.1186/1471-2458-9-359)
Supplement: Additional file 1 — Questionnaire. This file contains the questionnaire given to the students concerning antibiotic use. [file 1471-2458-9-359-S1.doc]

**Questionnaire**

**Aim:** This questionnaire is anonymous and is part of a study which aims to learn what students know about the use of antibiotics.

1. What is your age? I am _______ years old

Please answer if the following statements are true or false. Circle the correct answer.

2. Antibiotics are effective against:

Viruses True False

Bacteria True False

Fungi True False

Insects True False

Worms True False

3. Antibiotics should be prescribed for:

Colds True False

Influenza True False

Tuberculosis True False

AIDS True False

Hepatitis True False

4. Antibiotics should be taken with milk True False

5. Antibiotics do not interact with alcohol True False

6. Antibiotics can be taken at different times each day, if the daily doses are respected. True False

7. Antibiotic treatment should be stopped as soon as the patient feels better. True False

8. The incorrect use of antibiotics can lead to development of resistant bacteria. True False

Thank you for your participation.
